# Experiences of Hope after Treatment of Hepatitis C Infection—A Qualitative Study

**DOI:** 10.3390/ijerph192315732

**Published:** 2022-11-26

**Authors:** Knut Tore Sælør, Siv-Elin Leirvaag Carlsen, Lars Thore Fadnes, Lennart Lorås

**Affiliations:** 1 Department of Health, Social and Welfare Studies, Center for Mental Health and Substance Abuse, University of South-Eastern Norway, 3007 Drammen, Norway; 2 Department of Addiction Medicine, Haukeland University Hospital, 5021 Bergen, Norway; 3 Bergen Addiction Research, Department of Addiction Medicine, Haukeland University Hospital, 5021 Bergen, Norway; 4 Department of Global Public Health and Primary Care, University of Bergen, 5009 Bergen, Norway; 5 Department of Welfare and Participation, Western Norway University of Applied Sciences, 5063 Bergen, Norway

**Keywords:** hepatitis C, HCV treatment, hope, substance abuse, recovery

## Abstract

The aim of this study was to explore the experiences of hope people had after undergoing treatment for a hepatitis C virus infection (HCV). Between October 2019 and May 2020, 19 one-on-one interviews were conducted with people who inject drugs and had undergone HCV treatment. All participants had completed treatment and were documented as being virus-free. Data were audio recorded and then transcribed verbatim and analysed; a method inspired by reflexive thematic analysis. Those interviewed conveyed rich and nuanced descriptions of experiences of a life with HCV. Through the course of the analysis we developed four themes. The themes were formulated as metaphors aimed at capturing commonalities about how the participants seemed to “turn their gaze” after receiving HCV treatment: (1) turning their gaze backward; (2) turning their gaze inwards; (3) turning their gaze toward others and (4) turning their gaze forward. Participants’ descriptions of their experiences relating to HCV were somewhat gloomy, and HCV treatment seemed to inspire hope and a brighter outlook on several areas of life.

## 1. Introduction

Many of those struggling with substance problems experience co-occurring problems related to both mental and somatic health [1,2,3,4]. Among people who inject or have formerly injected substances, one of the most severe threats to their health is a hepatitis C virus infection (HCV), a blood-borne chronic infection [5,6]. HCV can cause liver cirrhosis and complications such as liver cancer or failure [7,8]. The prevalence of ongoing substance injection, and the transmission of HCV is high among people with opioid dependence [9,10], and almost 80 percent of HCV disease in high-income countries is related to drug injection [11]. Until recently about half of people receiving opioid maintenance therapy (OMT) in Norway had HCV [12], and liver disease is an major cause of death among people struggling with an opioid dependency [13,14]. 

Beyond a range of somatic symptoms and risks, HCV has also been reported to have psychosocial consequences [15,16,17,18,19]. A study found that participants successfully treated for HCV reported better overall health-related quality of life one year after their treatment compared to non-responders, despite the fact that a substantial number of participants continued injection use after successful treatment [20]. 

Until recently, key treatment for HCV was interferon-based, with moderate efficacy and considerable adverse effects, in addition to low treatment uptake among persons who inject drugs (PWID) [10]. In recent years, efficient direct-acting antivirals have also become available for PWID [21], yet, as with the older interferon-based treatment, direct-acting antivirals treatment coverage among people who inject drugs has, until recently, been low [22,23]. One of the measures aimed at increasing treatment uptake is establishing decentralized clinics focusing on interdisciplinary approaches, accessibility, and frequent follow-ups [24,25,26,27]. Such an approach is established in the two Norwegian cities of Bergen and Stavanger. Participants in the present study all receive OMT or low-threshold care in Bergen. In Norway, OMT includes supervised and observed intake of medications and frequent follow-ups by a multidisciplinary health care team that consists of nurses, social workers, peer counsellors, physicians, and psychologists, and is therefore a suitable arena in which to explore how HCV treatment is experienced [20].

### Hope and Recovery

The concept of recovery has gained a stronger foothold within the fields of substance abuse and mental health over recent years [28,29]. Within the field of mental health and substance abuse, recovery is often described as a personal and/or social process where the focus is on making it possible to live a meaningful life just as much as it is on eliminating the symptoms [28]. This might be perceived as standing in contrast to a more traditional bio-medical paradigm, where symptom reduction and clinical recovery is considered as a main goal [30]. 

Hope is central to overcoming a wide range of challenges and illnesses, e.g., [31,32,33,34], and despite the ambiguity of the term recovery, hope seems to be a common factor within the field of mental health and substance abuse [35,36]. 

HVC can have an impact on one’s quality of life and living conditions, as well as one’s outlook on the future. However, pure medical effects are often the main focus of investigation, and less is known on whether or not undergoing such treatment can change life perspectives and social roles. New treatment approaches are currently gaining foothold and are becoming more accessible. Exploring different dimensions of how new treatment methods affect recipients, and expanding knowledge through various methodological approaches, are important contributions in developing future practices. There is limited research on the influence of treatment of HVC on hope in the era of modern direct-acting antiviral HCV treatment. Thus, our aim is to explore participants’ experiences of hope after HCV treatment with direct-acting antiviral medications.

## 2. Method

The present study is part of a qualitative project aiming to explore how participants experience HCV treatment through focus groups and individual interviews. In the initial analysis of the data material, hope was an important topic that came up in the individual interviews. Hence, in this part study we have analyzed the individual interviews to explore how participants experiences of hope were influenced after HCV treatment. Qualitative methods aim to provide in-depth insights and are based on theories of human experience and are therefore well suited for this study [37]. As there is little research on the topic of our interest, we chose an explorative and interpretive design.

### 2.1. Recruitment and Details of the Participants

Our recruitment process was purposive and criterion-based, aimed to obtain participants that could contribute to the information needed for the project [38]. This entails that the participants were chosen based on the predetermined criterion or qualifications that are strategically relevant to the research questions and theoretical perspectives of the study [39]. None refused to participate, but due to the COVID-19-pandemic and related difficulties in some periods, a few possible participants were unavailable for interviews.

A total of 19 participants were interviewed individually. Among them were twelve men and seven women aged between 32–65 years. All participants came from Bergen. The participants had a range of 10 to 40 years of injecting substances. All participants had completed treatment and were reported as being virus-free. To ensure their confidentiality, we referred to them as ‘participants’ in the presentation of the findings. In the source population, most received social security benefits as main source of income, around one in six were homeless, and around half had less than 10 years of education [27], and many had a substantial burden of psychological distress [40]. The research nurses had some knowledge of the participants prior to inclusion linked to conducting health examinations.

### 2.2. Data Collection

Qualitative semi-structured individual interviews were used for data collection. The semi-structured interview examines themes that are largely decided beforehand, whilst still allowing the process and dynamics of the interview to determine how strictly the interviewer follows the script [41]. We involved user representatives in the development of the interview guide, and they assisted in testing questions with some peers. All interviews were conducted between October 2019 and May 2020 and transcribed verbatim. The participants in the individual interviews received a small amount of compensation for their time (NOK 200).

The interview templates for the individual interviews were co-constructed within the project group of the research project. The project group consists of researchers with broad interdisciplinary experience from research on substance use, welfare services, family therapy and professionals (social scientists, medical doctor, psychiatric nurses, and social workers).

The interview templates for the individual interviews focused on four main topics: (1) quality of life, (2) self-perception, (3) relations, and (4) substance use. For example, the participants were asked “How did treatment affect your everyday life?” and “Has the treatment affected your intake of substances when treatment was ongoing?” The interviews lasted between 30 and 50 min. The interviews were conducted by five research nurses involved in the project [20]. They all had extensive clinical experience from working with the target group, had been trained in qualitative interviewing, and were involved in developing the interview templates. All interviews were audio recorded, and transcribed verbatim.

### 2.3. Data Analysis

To generate a phenomenological and hermeneutical understanding of the participants experiences, we used reflexive thematic analysis, as described by Braun and Clarke (2019). Thematic analysis allows flexibility with regard to philosophical and theoretical stance. During the analysis we used Word for Windows to handle the data. The approach is a systematic, yet flexible, way of identifying and coding themes in qualitative data [42]. When conducting the analysis, we made some minor adjustments to Braun and Clarkes’ [42] approach ending up with the following four steps: (1) In this phase authors KTS and LL read and re-read the transcripts in order to familiarize themselves with the data material. Notes on tentative ideas and possible themes were taken and shared between the two of them. (2) In a bottom-up approach to data, LL developed preliminary codes and KTS revized, adjusted, and added codes to the transcripts. This allowed both authors to thoroughly re-read all of the interviews. (3) The third phase involved organizing possible themes based on the two authors understanding of the codes. This phase was initialized by LL and discussed and revisited in collaboration with KTS. The themes were then revised several times, before they were formulated into metaphorical terms, resembling what Braun and Clarke [42] refer to as a ‘latent approach’, which involves interpreting and exploring underlying meanings in data. (4) The final phase was writing this article, a circular process headed by KTS and LL with SELC and LTF contributing with both critical comments and contributions to the writing process.

### 2.4. Research Ethics

The study was conducted in accordance with the Declaration of Helsinki and approved by the Norwegian Regional Committees for Medical and Health Research Ethics (REK, 2017/51). All participants were informed about the option of withdrawing from the research project at any stage without the need for explanation, signed a consent form and received copies of the ethical approvals for the research project. Transcripts of the interviews were anonymized and stored on an encrypted server owned by the regional health authority.

## 3. Findings

Participants in this study shared stories of stigma and loss related to hepatitis C. Being aware that they had the disease gave rise to experiences of hopelessness and a lack of faith in the future. The interviews presented rich and nuanced descriptions of experiences from a variety of people, all with their own individual life story. Yet, through our analysis, we were able to develop four themes that seemed to capture some common and similar experiences described by participants in their interviews that were relevant to the focus of our study. One central common factor was how in a number of ways the participants seemed to experience HCV treatment as an opportunity to reorient, and to some extent influence how they perceived both their understanding of both their past and future. With the aim of capturing and to some extent abstracting the rich material, the themes were formulated as different metaphors revolving around how the participants seemed to “turn their gaze”: (1) turning their gaze backward; (2) turning their gaze inwards; (3) turning their gaze toward others and (4) turning their gaze forward. Common to all the themes is that they involve “turning” the participants’ gaze in various ways. Though they should not be understood literally, they nonetheless capture experiences of the reorientation towards and hopes for the future that were conveyed in the participants’ stories.

### 3.1. Turning Their Gaze Backwards

The participants described being infected with HCV as having the “addict’s disease”. If you told anyone about the illness you were automatically labelled a junkie. One participant said that: “By turning yellow you are branded as having the junkie disease”, referring to yellow tanning related with advanced chronic liver disease. Another participant stated that “when you have hepatitis C, people know that you are an addict”. Being “branded” was a description several of the participants used. “The colour yellow” was perceived as an obvious sign of who they were, and what kind of life they had lived. As “branded”, they were afraid of being outcast, both by others who use drugs, and by “significant others” who are not part of an illicit drug scene. The fear of being an outcast made several of the participants keep the infection a secret. When in contact with public health services, however, the diagnosis was noted and known by those involved. Because of this, many of the participants felt “branded” and considered as an infectious “junkie”. They experienced this, for instance, as there being a lack of respect for them as a person and a lack of knowledge about hepatitis C. One of the participants revealed that a doctor had refused to offer him treatment. The participants ascribed this as a combination of being part of a stigmatized group, and an obvious lack of knowledge among health workers and the general public on how hepatisis C transmits. As a person who was infected, they had all experienced shame. The shame was experienced not only in encounters with the general public, but also within the social and cultural environment of people who inject drugs. Solely by being a person having a substance abuse problem, they were already part of a stigmatized group. Being infected with hepatitis C, they were at the lowest step of the “social ladder”, including among those who they considered as their peers. They were stigmatized in an already stigmatized group. This confirmed the negative thoughts participants already had about themselves and led them down a destructive spiral were they felt that they had little reason to live. Hopes for the future were diminished, as one participant put it: “You do not have much to live for. Your mind is set on the next fix, you no longer have any visions for the future”.

Participants shared stories of stigma involving encounters with both the general public and persons who use illicit drugs, and what may be defined as internalized stigma. One participant said: “It’s just like the word ‘addict’ is inscribed inside me”. Most participants described being infected as making them feel nasty and filthy, something they wanted to distance themselves from. Yet, considering hepatitis C is potentially lethal, the result was that initiating changes seemed pointless since they were bound to die at a young age regardless. Being “dirty inside, yellow and identified as sick” by society became part of a negative identity. A negative identity which was constantly confirmed through stigmatization and being rejected by acquaintances in the illicit drugs scene, professional helpers, and “significant others” “outside of the drugs scene”.

### 3.2. Turning Their Gaze Inwards

The participants described the experience of being prioritized to receive the costly HCV treatment as giving them a sense that had worth as a person. Their interactions during the treatment were also described as very positive. They were treated respectfully, and treatment was adjusted according to their needs. Mastering the treatment influenced their perception of themselves in a positive way. A participant described that “mastering the treatment gave me faith that it’s possible to overcome trouble in life”. This notion of challenges being manageable made the participants believe that they could influence their future. This, in turn, inspired participants’ sense of hope.

Through their treatment, recipients obtained new knowledge about HCV and how the infection spreads. Some participants in our study had passed this knowledge on to others in the illicit drug scene. For some, this had altered their roles, from being stigmatized in a stigmatized group, to being regarded as a resource, all of which improved their perception of themselves. This improved self-perception also made them more aware of their own health. No longer did they perceive themselves as “dirty on the inside”. They were more aware of the ways in which everyday decisions influenced their health. Despite the HCV treatment not being considered a substance abuse treatment, it changed participants’ view on their ability to control their substance use. Even though substances were still part of most of the participants’ lives, their attitudes to substance use had changed. Being what they described as a “worn out and tired drug addict”, they had not been watchful to their intake of substances. As one participant said: “If you’re worn out, you do not give a shit about anything, including infections”. Now that they had hopes of an improved future, they no longer wanted to share drug-taking equipment. Participant 6 said that “after I got well, I quit sharing equipment and became very cautious about hygiene”. The participants were also conscious with regard to buying their substances from the safest source possible, something which few had cared about before treatment. In general, the majority also declined offers of substances to a greater extent. This was an obvious step toward setting limits on their own behalf. As one participant put it: “I am more conscious about how far I go in terms of putting pressure and expectations on myself. Now, if I experience a negative thing in life, I feel I can weed it away”.

### 3.3. Turning Their Gaze toward Others

When successfully treated for HCV, they no longer felt “marked” by the “junkie disease”. Furthermore, shame, guilt, and experiences of stigma seemed to diminish. The experience of getting well was of great importance to how they perceived themselves and how they interacted with others. Through seeing themselves more positively, they gradually saw their self-confidence, courage and feeling of energy to act in life improve.

Improved self-confidence and not being infectious gave the participants an opportunity to reconnect with significant others. Many had felt excluded and unwanted when infected with HCV. Experiences of not getting to spend time with family members such as grandchildren, or not being allowed to use the toilet when vising their family, underpinned the feeling of unwantedness and that others were afraid of infection.

After recovering, several participants had experienced greater awareness and reflexivity related to how their behavior influenced their interactions with others. These changes were also noticed in their surroundings, such as with their family and other significant others, and one of the participants stated that: “My family has said that I seem more confident in myself”. Another participant spoke about changes in their use of language: “The language, ethical language. It flies out the window when you do not feel good about yourself”.

Spending time with significant others, like family and friends, led to an upward spiral of encouragement, as one participant put it: “It’s always nice to meet new people. The way they treat you is feedback in itself”. Positive feedback challenged how they had previously perceived themselves as persons with substance abuse problems with little hope for the future, a perception which may be interpreted as internalized stigma. Positive feedback, in turn, gave them the confidence to seek and establish acquaintances beyond their “inner circle”. Through such experiences when meeting new people, the participants felt that they were being acknowledged in a way that they described as “normal”. Along with improved self-esteem, their raised consciousness about their own actions, and the fact that they were no longer contagious, made it possible to initiate intimate relations. Shame, stigma, fear of contagion, and/or rejection, had resulted in some of the participants avoiding intimate relations while having HVC. One participant described it like this: “Intimate relations and having sex was not considered possible when being ill [with hepatitis C]”. In addition, impaired bodily functions made it difficult to participate in sexual relationships. The participants experienced opportunities for intimate relationships due to improved health and reduced impairments. Furthermore, it made it easier to relax, feel confident, and approach others, as one participant said: “In sexual relations, I can now relax and feel safe that I do not need to use a condom”. People in established relationships, described feeling closer to their partners after successful treatment of hepatitis C. Another participant said that: “Our relationship has become more mature”.

### 3.4. Turning Their Gaze forward

Since reaching old age was regarded as unattainable, changing behavior viewed as unhealthy was perceived as meaningless when infected with HVC. This changed after successful treatment. One participant stated that: “It feels good to know that is not the [hepatitis C] disease that will end my life”. Participants were now less concerned about dying from a substance related disease. Their thoughts on who they were and how they would be remembered were now altered.

As death was no longer considered imminent, it was possible to look forwards. The future was now viewed in a more hopeful manner. A hope that change and a better life were possible. Hope also made it possible to face problems which in the past had affected their mental health and finances, but at earlier times seemed unfeasible. As expressed by one of the participants: “After the treatment [for hepatitis C], I can look forwards. It is possible to work on my own mental health and participate in treatment programmes”. While some initiated treatment and follow-up of somatic disease and mental health problems, others experienced that their problems vanished after their HVC treatment. For some, getting well resulted in an improved ability to concentrate and higher levels of energy, which made it possible to fill their lives with meaningful activities, which in turn enhanced their mental health. Participants described bodily changes, such as less sweating, and improved tolerance for physical activity. As one said: “I think of it like ripples in the water, positive changes lead to more positive changes”.

After experiencing that their mental and somatic health problems had lessened, some of the participants described their thoughts as focusing less on substance use and more on building a future. They also expressed feeling acceptance of their current situation and its opportunities. Many participants described using substances less and that they could do more work that contributed to improving their financial situation that for many had been difficult for years. As stated by one participant: “Now we have money to get through the month. We can even put some money aside. Earlier, we only waited for an imminent death”.

## 4. Discussion

The presentation of our findings may give the impression that “turning your gaze” is straight forward, and that treatment represents a linear, tidy, and neat journey. That is not our intention. Life is rarely linear and tidy, and stories of hope or recovery are certainly not [35].

Hepatitis C differs from mental health problems and substance abuse in many regards. Yet, their interconnectedness is apparent, including for those participating in the present study. Our aim was to explore experiences of hope after receiving HVC treatment. The stories that the participants shared may seem embossed with despair, yet they carry testimonies of hope as well. Weingarten [34] puts forth her construct of ‘reasonable hope’ as a variant of hope. In contrast to many of the traditional images of hope, which often come in black and white categories, where hope and hopelessness are opposite, reasonable hope accommodates despair, contradictions, and doubt. Reasonable hope is humble and directed toward what is within reach. It is not something you either have or do not, something you feel or not, but a practice, and something you do with others; it is relational. Despite the fact that the future is uncertain, reasonable hope maintains that it is possible to influence it, and one way of practicing reasonable hope is identifying realistic goals and pathways towards them. Weingarten’s approach to hope seems to resonate with the findings of our study, stories that entailed despair, uncertain futures and a need to set goals on the way forward.

Biong and Herrestad [43] describe hope as: “… opening something which is locked” (own transl.) (p. 50). For participants in the present study, it somehow seemed to be the other way around: treatment appeared to unlock hope. It made the future appear brighter, more hopeful, and gave rise to both opportunities and actions aimed at enhancing participants’ lives.

Within the field of mental health and/or substance abuse, *clinical* recovery is often contrasted to personal and social recovery and deemed the hegemony of medicine and its experts [28,44]. When seeking health services, people, along with the challenges they might experience, are at times seemingly split into parts. This often results in fragmented services that do not meet their needs. For people seeking support, fragmented services and bureaucratic thresholds may diminish hope [45]. Richmond et al. [46] explored how successfully treating hepatitis C influenced peoples’ lives. Treatment reduced uncertainty and fear about the future. Furthermore, it allowed participants to clarify what physical effects could be attributed to hepatitis, and what could be attributed to other health problems. The authors also state that: “Most significant was the psychological impact of being cured, highlighting the serious impact of hepatitis C and drug use related-stigma on the lived experience of people with hepatitis C and the uncertainty related to the physical impact of living with the infection” [46] (p. 7). For those participating in our study, what may be labelled as psychosocial consequences caused by a viral disease appeared just as difficult to cope with as physical symptoms. Undoubtedly, physical symptoms and an outlook of a premature death, treatment for the viral infection, along with reducing physical impacts, was important. Nevertheless, stigma and the effects it had on participants’ lives seemed just as detrimental. Similarities with the field of mental health is striking, where rebuilding an identity that goes beyond ones’ diagnosis is often a key factor in recovery [47,48]. When conceptualizing recovery from co-occurring mental health and substance use problems, Davidson et al. [35] list *understanding and accepting self* as central for recovery (p. 8), along with renewing hope, confidence, and commitment [35]. Drawing on our findings, treatment for hepatitis C seemed to influence all of the above. What may be labelled as *clinical* recovery somehow sparked what often is categorized as personal and social recovery [28,44]. The notion of recovery is highly debated [49], and our ambition is by no means to provide what we regard as the right answer. Nonetheless, in relation to hepatitis C and substance (ab)use, dividing people, along with their challenges, hopes, or recovery, into silos according to organizational issues or needs seems futile.

### 4.1. Code Yellow

Based on our findings, there are several parallels between how hepatitis C impacts peoples’ lives, and experiences of mental health and substance abuse problems. Among them are stigma and the lack of comprehension that often goes hand in hand, both in the general public and within professional services, e.g., [45]. For those interviewed in our study *the yellow colour* was literally comprehended as a brand, signalling that you were “an addict” or a “junkie”. This is in line with Butt [50] who wrote: “The intrapersonal or individual dimension may be experienced through internalization and acceptance of society’s views. For example, the internalization of oneself as an infectious agent may lead to self-descriptions of being ‘dirty’ and ‘diseased’” (p. 715). Furthermore, participants in our study highlighted that being contagious and filthy became a part of their identity and made them feel stigmatized within an already stigmatized group.

When seeking professional help, participants had received substandard treatment because of what they referred to as *the yellow colour*. Not only did they ascribe this to a lack of respect, but also to a lack of knowledge among professionals. Such negative experiences related to stigma and HVC are by no means unique to our study, but in line with what Butt [50] has found. Exploring life with hepatitis C, Dowsett et al. [16] found that many had negative experiences with healthcare providers. Being treated insensitively, a lack of communication, insufficient time spent with them, professionals focusing on the disease rather than the person, feeling unworthy of care or being denied treatment at all, were examples of such negative experiences. This might seem dismal, and the authors stated that: “Very few of these study participants with HCV reported positive experiences with the healthcare system” (p. 8). Stigma was a common experience which had a significant impact on people living with hepatitis C, and fear of transmission and association with injection drug use and risky behaviours were two contributing factors to HVC related stigma [16]. Some of the reactions to this stigma included shame, embarrassment, isolation, low self-worth and feeling dirty. The authors behind the study suggest compassion and education as being among several countermeasures to this [16]. Our aim is not to devalue health care, yet it is striking that a lack of knowledge and negative attitudes among health care professionals hinder people from seeking treatment for a viral infection. Stigma and substandard treatment caused by a status of being “an addict” is by no means acceptable and may diminish peoples’ hopes for the future [45,51].

### 4.2. Blending in

For persons having experiences that might be considered extraordinary, and life stories embossed by defeat and disappointment, what can appear mainstream may represent something to aspire to and the idea of becoming a part of it may inspire hope. What is considered mainstream is as diverse as life itself, yet a sense of security and belonging seems central [52]. One way of understanding recovery is “being part of a fellowship” (own translation) [53]. Experiences of exclusion made the future appear bleak to those participating in our study. Some participants had experienced that stigma associated with HCV resulted in exclusion and not being able to nurture relationships with significant others. *The yellow colour* had become part of the participants’ identity and getting rid of it was literally a means of altering ones’ identity, away from someone associated with injecting drugs, toward what several of the participants described as *normal.* Participants in the above-mentioned study by Richmond et al. [46] expressed that when no longer infectious, they “[felt] normal” (p. 3/9) which had a positive impact on relationships with their family or others they were close to, and when seeking professional health care. Several of the participants who had stopped injecting stated that treatment reduced guilt and shame caused by their previous lifestyle: “It’s like the last mark … is now gone. All the rest is just memories that I don’t have to think about” [46]. The similarity with our findings is striking. As the yellow colour disappeared, the future appeared brighter and more hopeful. As Richmond et al. [46] describe, it is like “breaking a connection to the past” (p. 4). Singh et al. [54] explored how treatment with direct-acting antivirals was experienced among 25 persons with an active HCV infection and found that participants experienced physical and mental health problems prior to treatment. HCV affected participants’ relationships with friends, family, and sexual partners due to a fear of transmitting the infection and stress related to this. After treatment, participants expressed having received a “new beginning”, increased self-esteem and self-worth, and positive changes which included reduction in substance misuse [54].

Despite the conception of reasonable hope as relational, Weingarten [34] makes it clear that: “not all relationships will give rise to or support reasonable hope” (p. 8). Participants in our study shared stories of discouraging encounters with health professionals. Additionally, their relationships with friends and family were also influenced in negative ways. After treatment, this seemed to change as treatment somehow opened doors and gave rise to opportunities long put on hold. It enabled participants to expend their social network and maintain existing ones. No longer burdened by *the yellow colour*, their self-esteem improved, and they were more sensitive to how they interacted with, and influenced, others. Belonging to new fellowships seemed more within reach.

### 4.3. Connecting the Dots

Hope does not entail certain outcomes, and hope is influenced by the past, yet oriented towards the future [52,55]. Often, people tend to gaze back and linger on the past, wishing that circumstances could somehow change from what they once were, at least that is how hope is sometimes expressed. However, there is no such thing as returning to what once was, the only thing that returns is difference [56]. Weingarten [34] believes that reasonable hope resembles the chaos and unpredictability that is often a part of life: “The line drawn to depict a reasonable hope narrative does not necessarily go straight from bottom to left to top right. A variety of lines may represent a reasonable hope narrative because it is the activity of making sense of what is happening to us, not an outcome, that is the heart of reasonable hope” (p. 8).

Koehn and Cutcliffe [57] described inspiring hope as part of substance use treatment in three phases. One of these phases is linked to the self, relationships, and the future, and is described as “shaking up” their current way of being and “relating to the world” (p. 86). This includes reclaiming a sense of personhood, facilitating relationships with others and embracing possibilities for an altered future. Weingarten [34] emphasises that: “reasonable hope’s objective is the process of making sense of what exists now in the belief that this prepares us to meet what lies ahead. With reasonable hope, the present is filled with working not waiting; we scaffold ourselves to prepare for the future” (p. 7). Weingartens [34] construct of reasonable hope does not focus on achieving goals, but on how one can orient themselves towards them. Reasonable hope is about making sense of what happens to us, not focusing on positive outcomes, and that it: “does not struggle against an uncertain, unknowable future, but rather embraces it as its best bet. In dire circumstances (…) it is precisely because we cannot know what the future may bring that using reasonable hope (…) helps us toward something better than what we are living now” (pp. 8–9). Mattingly [58] states that many of the narratives about hope from the field of medicine are expressed in ways resembling a war. The doctor or patient fights the disease, or enemy, and is victorious in the end. This has similarities with how recovery may be perceived from this perspective. Mattingly [58] underscores that it could be fruitful to think about hope as hard work. Problems rarely vanish by themselves, and treatment of hepatitis C is no miracle cure, but it seems to have been essential for many participants in our study—far beyond how it is often described by medical practitioners.

Participants in the present study had achieved one important goal: treatment for hepatitis C. In hindsight, or when analysing qualitative data, it sometimes seems straightforward to identify themes, patterns, or somehow connect the dots. In the midst of a chaotic life-situation, this is seldom the case. We are in line with Davidson et al. [35] who wish to: “avoid the circuitous proverbial question, ‘which came first, the chicken or the egg’” (p. 285) when portraying experiences of hope or recovery. Still, it seems that treatment had a great influence on participants’ lives. If our goals seem too distant, too hard to reach, and hard to imagine, things may appear as stuck and our outlooks become hopeless [34,43]. For participants in the present study, the cure for what is strictly a viral disease also made it possible to act on mental health and financial problems. Doing reasonable hope can involve identifying realistic goals, along with plans on how to achieve these [34]. In all likelihood, most of what followed from treatment for hepatitis C could not be foreseen or planned upfront. Despite it not being a goal, treatment gave an incentive to be more caring about oneself, and reduced intake of substances. Having hopes for the future seemingly made them more aware and cautious of the risks associated with injecting drugs. Not everybody who uses various substances aims to reduce their intake, yet several participants in our study expressed that they had as a consequence of their hepatitis C treatment. This corresponds well with prior research [15]. Based on our findings, treatment for hepatitis C seemed to start what may be characterized as a domino effect.

## 5. Limitations

Searching for patterns and connections in the data reduces complex and rich stories into overarching and common themes. We do not claim that these are valid across what may at first glance seem like similar situations, nonetheless they convey parts of stories we consider important. Rather than representing experiences, our findings may contribute to discussions and practices related to hepatitis C treatment and the experiences that may go hand in hand. Despite our efforts to conduct a credible and transparent analysis of data, thematic analysis has its weaknesses, and applying the criteria described by Nowell et al. [59] could have contributed to the trustworthiness of our findings. One obvious limitation of this study is that interviews were to some extent conducted by nurses who were known to participants as health professionals from clinical settings and for treatment of hepatitis. This may have led to participants putting constraints on themselves in regard to negative experiences related to the treatment, and a desire to present a positive point of view. Having conducted the interviews ourselves, would naturally have influenced the results. Other analytical approaches, follow-up interviews, or a quantitative approach could certainly yield interesting findings. It is important to note that the included participants were all treated with modern direct-acting antiviral medications, that are generally highly effective and well tolerated [60]. The experiences could probably have been different if patients had received the formerly used interferon treatments, that were both less effective and were linked to substantial side effects [60].

## 6. Conclusions

For those participating in this study, life had at times involved bleak future outlooks, or seemingly no future at all. Receiving treatment for hepatitis C was by no means a quick fix regarding complex and intertwined challenges that can go hand in hand with illicit drug (ab)use. Treatment gave rise to hopes of a more positive future. Much like treatment, hope is no quick fix, but should not be underestimated. The ripple effects that treatment seemed to have on participants lives were striking. Not only had treatment affected participants’ health in numerous ways, but it also influenced how they perceived their interactions with others and how they were treated differently by their surroundings. The consequences of stigma and sub-standard treatment relating to hepatitis C cannot be underestimated, and yet it seems that counteracting such devastating consequences must involve societal factors alongside health care services. Implications from this study include that diverse and multidisciplinary approaches are likely to be beneficial for persons struggling with substance related problems and HCV. Further, treatment for HCV affects health in a broad sense, and succeeding in meeting the target group’s needs is of great importance. The socio-economic gains should not be undervalued, neither should the positive effects of strengthening and reestablishing relations with loved ones. This further adds to the importance of scaling up treatment of hepatitis C.

## Data Availability

The data generated and analyzed during the current study are not publicly available due to national ethical regulations.
